# Sigmoid Diverticular Stricture With Large Bowel Obstruction, Hepatic Flexure Perforation, and Nonfatal Retroperitoneal Necrotizing Fasciitis Extending to the Neck

**DOI:** 10.7759/cureus.92191

**Published:** 2025-09-13

**Authors:** Mohammad Shiraz, Mohammad A Waseq Hossain, Elisabeth Drye

**Affiliations:** 1 General Surgery, Peterborough City Hospital, North West Anglia NHS Foundation Trust, Peterborough, GBR

**Keywords:** bowel obstruction, bowel perforation, necrotizing fasciitis, sigmoid diverticular stricture, total colectomy

## Abstract

The most prevalent type of colonic diverticulum is sigmoid diverticulosis. Nevertheless, complications such as stricture formation, potentially leading to intestinal obstruction, are quite rare. Another uncommon consequence is perforation, which can, in exceptional cases, result in necrotizing fasciitis; however, spread to distant fascial planes is extremely unusual.

We present a remarkable case of a 61-year-old man who presented with sigmoid diverticular stricture causing large bowel obstruction and perforation. This resulted in extensive necrotizing fasciitis tracking from the retroperitoneum through the chest and into the cervical region. Despite the severity, the patient survived after undergoing an emergency laparotomy, total colectomy, serial debridements, and negative pressure wound therapy. This case highlights a unique complication of diverticular disease with an exceptionally favorable outcome.

## Introduction

The abnormal protrusion of a sac-like bulge into the sigmoid colon wall is known as a sigmoid diverticulum. While a diverticulum can develop anywhere in the colon, it most frequently occurs in the sigmoid colon. Uncommon complications include stricture formation leading to perforations in sigmoid diverticulosis. Like the study by Nagorney et al., which described perforation leading to generalized peritonitis, tracking to distant fascial planes is very rare but can result in necrotizing fasciitis [1]. When necrotizing fasciitis spreads from the retroperitoneal to the cervical areas, it causes a severe infection of soft tissues and increases the risk of death. This case demonstrates that diverticular disease can cause atypical, life-threatening complications, highlighting the need for early imaging and a staged surgical approach.

## Case presentation

A 61-year-old man who had been complaining of right-sided abdominal pain for the past three days presented to us at the hospital. Over the past three weeks, he had been experiencing pain, decreased hunger, and an overall reduction in weight. The patient did not mention any episodes of nausea, vomiting, or fever. He denied ever having abdominal surgery or having been diagnosed with any gastrointestinal disease, and he gave no other significant medical history. The patient's systemic review was unremarkable, and he had no additional complaints. No history of previous hospital visit was reported by the patient for this condition.

Upon general physical examination, the abdomen exhibited diffuse tenderness upon palpation, with the most significant tenderness and localized guarding observed in the right lower quadrant. The examination revealed the absence of a palpable mass; however, the abdominal wall exhibited tenderness and mild distension. Upon thorough inspection and palpation, no groin hernia was detected.

**Figure 1:**
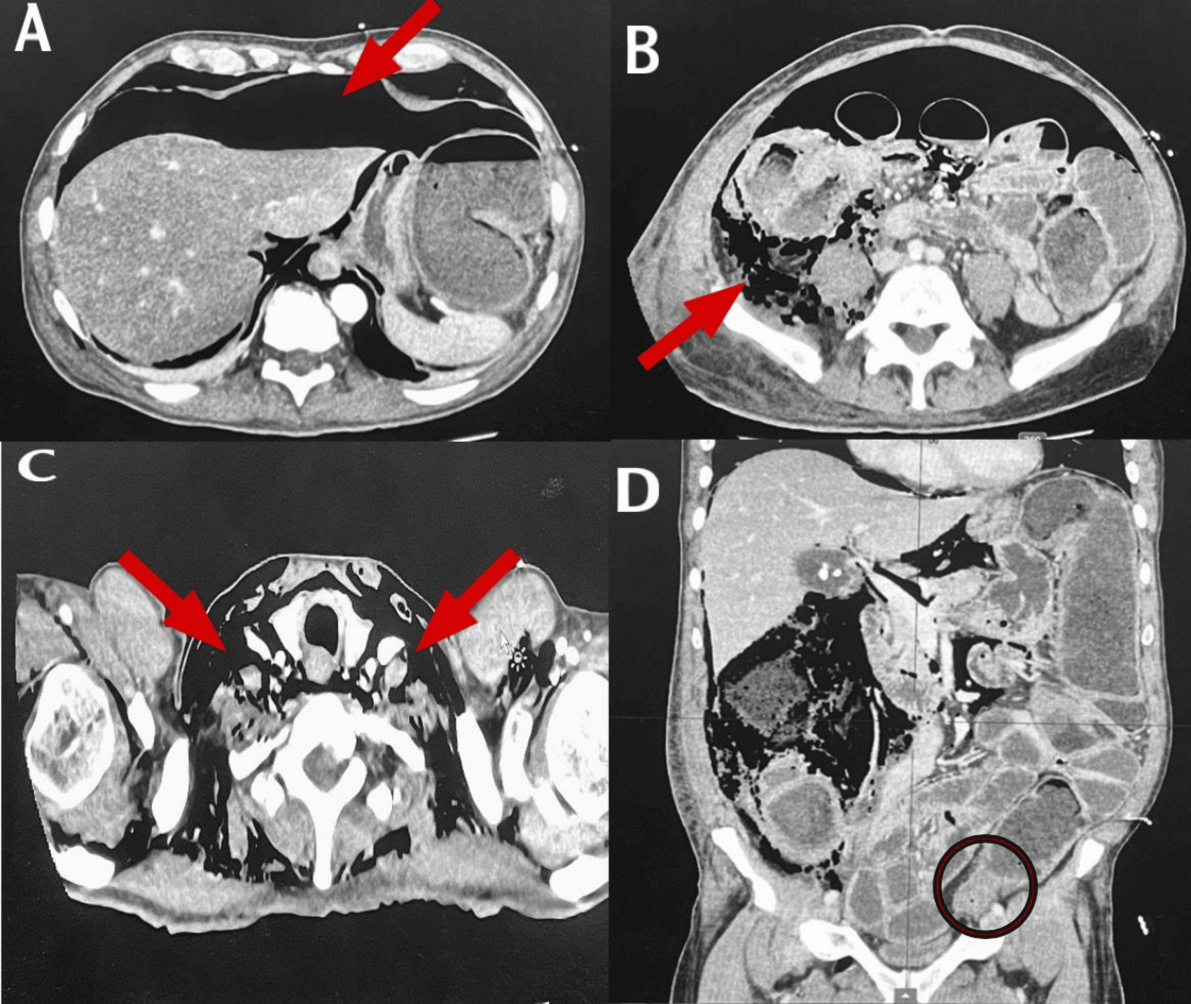
Computed tomography of the thorax, abdomen, and pelvis: (A) extensive pneumoperitoneum, (B) right retroperitoneal extension, (C) pneumomediastinum and extension in the neck, and (D) sigmoid stricture causing bowel obstruction

As shown in Table 1, laboratory investigations were done, which showed raised C-reactive protein (CRP) and reduced estimated glomerular filtration rate (eGFR). Upon radiological investigation, a chest X-ray showed air under the diaphragm. As shown in Figure 1, a computed tomography (CT) scan of the thorax, abdomen, and pelvis was done on day 1, which showed features of pneumoperitoneum extending to the right retroperitoneal space and right lumbar region. The CT also showed pneumomediastinum and surgical emphysema in the upper chest wall and lower neck, appearing secondary to perforation of the obstructed bowel with a transitional point in the sigmoid colon. There were features of an underlying stricturing mass lesion in the transitional zone in the sigmoid colon affecting a segment about 73 mm long. There was diffuse thickening of the distended colon wall.

**Table 1:** Laboratory investigations on admission and during hospitalization WBC: white blood cell; CRP: C-reactive protein; eGFR: estimated glomerular filtration rate

Lab parameters	Day 1 (admission)	Day 3	Day 7	Reference
Inflammatory markers				
WBC (×10³/µL)	9.8	12.5	10	4.5-11
CRP (mg/L)	379	185	153	<5
Renal function				
eGFR (mL/min/1.73 m²)	46	62	82	>90
Full blood count				
Hemoglobin (g/dL)	14.2	12.3	11.9	12-16
Platelets (×10³/µL)	380	320	345	150-450
Metabolic				
Lactate (mmol/L)	6.46	-	-	0.5-2.2

On day 1, the patient was taken to the operating theatre, and he underwent an emergency exploratory laparotomy. During the surgical procedure, observations indicated a perforation located at the hepatic flexure, accompanied by fecal contamination within the peritoneal cavity. The colon exhibited significant dilation and distension proximal to that of the obstruction. A significant stricture was observed in the sigmoid colon. Initially, the stricture felt malignant rather than being due to diverticular disease. The pathophysiological link between the distal sigmoid stricture and the proximal perforation was by upstream barotrauma as per Laplace's law rather than ischemia. Proximal to the obstruction, luminal pressure caused massive colonic distension, significantly increasing the radius. This led to a critical increase in wall tension, overwhelming the tissue elasticity and ultimately resulting in blowout perforation at the site of greatest diameter, in this case, the hepatic flexure. Moreover, substantial necrosis was observed, accompanied by a purulent collection centralized in the right paracolic gutter. A total colectomy via open approach was done, and total colectomy was preferred rather than segmental resection due to the considerable degree of colonic damage and contamination seen in the patient. Debridement of the necrotic tissue found in the right paracolic gutter was performed. Following this, a negative pressure dressing, AbThera (Solventum, Saint Paul, Minnesota, United States), was inserted into the wound as the plan was to bring the patient back in 24-48 hours to reassess the area of necrosis. The patient was moved to the intensive care unit (ICU) and was kept intubated and on inotropic support.

On day 2, the patient remained intubated and on inotropic support.

**Figure 2:**
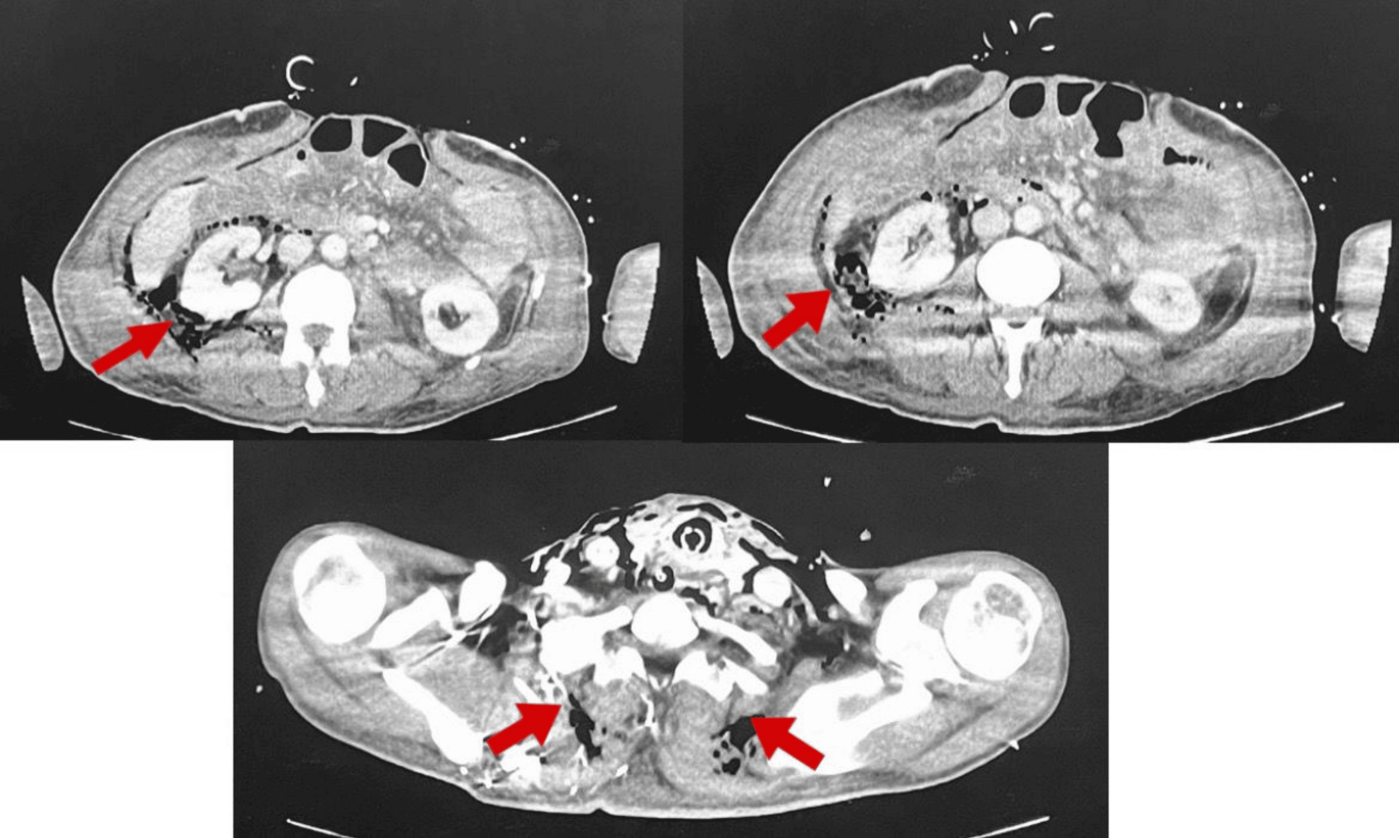
Computed tomography of the thorax, abdomen, and pelvis done on May 5, 2025, showed a significant reduction in the pneumomediastinum, surgical emphysema, and pneumoretroperitoneum

On day 3, a second laparotomy was performed. Further debridement of the necrotic tissue in the retroperitoneum was required, and a peritoneal washout and replacement of the AbThera VAC dressing were performed. As shown in Figure 2, a repeat CT of the thorax, abdomen, and pelvis was done the following day, which showed a significant reduction in the pneumomediastinum. Mild to moderate bilateral pleural effusions and mild ascites were seen in this CT. Surgical emphysema, pneumoperitoneum, and pneumoretroperitoneum were also reduced. The third laparotomy, which was performed on day 5 as part of the planned re-evaluation, confirmed the viability and health of the bowel.

The patient was maintained on low-dose inotropic support, demonstrating stability and indications of clinical improvement, as evidenced by an increasing reduction in CRP levels. Extubation was successfully performed on day 7. Subsequent days revealed the emergence of a high-output stoma; however, the patient maintained a positive trajectory in recovery, aided by continuous supportive care. He was discharged home after 25 days, looking after an ileostomy. The postoperative histology showed perforated diverticulitis with no malignancy.

## Discussion

Sigmoid diverticulitis is a condition that is prevalent in industrialized nations and is becoming more common and expensive. As documented by Stollman and Raskin, the United States saw a 29% rise in elective surgeries and a 26% rise in hospital stays for diverticulitis between 1998 and 2005 [2]. Diverticular illness is common in Western nations, especially among people over 60, and most people with the condition don't even know it's there. Inflammation, hemorrhage, perforation, fistula development, and stricture are all possible consequences; the latter is less prevalent but still has important clinical implications. Progressive luminal narrowing, which can occur as a result of sigmoid strictures caused by chronic diverticulitis, puts the patient at risk of developing a major obstruction of the bowel. As described by Stollman and Raskin, perforation at the hepatic flexure in this patient occurred as a consequence of the unusual but serious complication of intestinal distention leading to upstream perforation [2]. As documented by Slam et al., this case demonstrates the application of Laplace's law where a sigmoid stricture creates a closed loop obstruction leading to dilatation and a massive increase in the radius of the proximal colon, with the wall tension being highest at the point of greatest radius, i.e., the hepatic flexure, directly explaining the site of perforation despite pathology originating distally [3].

As demonstrated in the case by Secil and Topacoglu, the presence of pneumoperitoneum, pneumomediastinum, and retroperitoneal air indicates that intraluminal gas has been widely expelled via the perforation site; in such instances, the infection might progress retroperitoneally and, on occasion, follow fascial planes [4]. This case parallels the imaging findings of Kawamoto et al. Despite a fulminant necrotizing fasciitis that spread with remarkable aggression from the right paracolic gutter, through the chest, and up to the neck, the patient survived. This rare and extensive dissemination highlights the critical need for the prompt and radical surgery that was performed [5].

As illustrated in the case report by Giri et al., acute diverticulitis can produce relative luminal constriction due to pericolic inflammation and abscess formation, which can lead to partial colonic blockage; additionally, colonic pseudo-obstruction may occur [6].

An infection of the soft tissues known as necrotizing fasciitis can quickly worsen and perhaps kill the patient. Retroperitoneal necrotizing fasciitis due to bowel perforation is extremely rare and frequently deadly, especially in cases when debridement as well as diagnosis is postponed. As Stocchi notes, while this condition is more prevalent in the perineum and its surrounding area, its occurrence in the retroperitoneum signifies a severe and atypical progression [7].

Both the diagnosis and the monitoring processes relied heavily on radiological imaging. The early CT results were instrumental, revealing obstructive pathology, perforation, and the critical extension of extraluminal gas along fascial planes, a crucial indicator of necrotizing fasciitis that prompted immediate surgical intervention. This case underscores the necessity of meticulously evaluating for extra-abdominal gas, such as subcutaneous emphysema, pneumoretroperitoneum, or pneumomediastinum, whenever extensive pneumoperitoneum is present, as it often signifies a more severe, spreading infectious process. To help evaluate the efficacy of the intervention, follow-up imaging verified that the pneumomediastinum and pneumoperitoneum had partially resolved.

In older patients having a previous history of diverticular illness, it is crucial to keep a high suspicion for bowel blockage due to strictures, particularly in cases where systemic toxicity is evident. This case highlights the necessity of this approach. The significance of rapid imaging and surgical investigation should not be underestimated when dealing with extensive gas dissection, as it can signal the beginning of necrotizing fasciitis.

This case's conclusions are drawn with the acknowledgment of certain limitations, primarily the absence of preoperative colonoscopy or biopsy to definitively characterize the stricture and the reliance on postoperative histology to exclude an underlying malignancy.

In summary, this case underscores that a rapidly deteriorating clinical course in the context of a known diverticular stricture mandates urgent surgical intervention. Furthermore, the presence of extensive subcutaneous emphysema should be recognized as a critical red flag for necrotizing fasciitis, a surgical emergency that requires immediate and aggressive management.

## Conclusions

A rare but devastating consequence of diverticular disease is sigmoid stricture, which causes large bowel blockage, upstream colonic perforation, and retroperitoneal and neck necrotizing fasciitis. Sigmoid strictures are an important cause of large bowel obstruction in older patients with diverticulosis. Unusual radiological signs (e.g., pneumomediastinum, subcutaneous emphysema) should raise the alarm for retroperitoneal perforation and fascial tracking. Retroperitoneal necrotizing fasciitis is a surgical emergency requiring immediate exploration and planned re-look laparotomies. The absence of malignancy on histopathology highlights the aggressive potential of diverticular disease itself, reinforcing the need for extreme vigilance in patients with known diverticulosis who present with obstructive symptoms and systemic toxicity. These life-threatening sequelae demand a low threshold for advanced imaging and prompt surgical exploration, as early recognition and intervention are critical to mitigating their rapid and devastating progression. The rapid progression of necrotizing fasciitis in this case underscores the importance of early imaging, particularly CT, to identify perforation and tract infection spread. Additionally, the successful management of this patient relied on a multidisciplinary approach combining emergency surgery, intensive care support, and repeated reassessment.
